# Identifying the trends in wound-healing patents for successful investment strategies

**DOI:** 10.1371/journal.pone.0174203

**Published:** 2017-03-17

**Authors:** Jae Ha Gwak, So Young Sohn

**Affiliations:** Department of Information and Industrial Engineering, Yonsei University, 134 Shinchon-dong, Seoul, Republic of Korea; University of Pisa, ITALY

## Abstract

**Background:**

Recently, the need for rapid wound-healing has significantly increased because of the increasing number of patients who are diagnosed with diabetes and obesity. These conditions have contributed to a surge in the number of patients with chronic wounds worldwide. Furthermore, many cost-effective wound-healing technologies have been developed in order to keep up with the increased demand. In this paper, we performed a quantitative study of the trends associated with wound-healing technologies using patent data.

**Methodology:**

We analyzed the trends considering four different groups of patent applicants: firms, universities, research institutes, and individuals using a structural topic model. In addition, we analyzed the knowledge flow between patent applicants using citation analysis, and confirmed the role of applicants in the knowledge-flow network using k-means clustering. As a result, the primary wound-healing technology patents applied for by the four groups varied considerably, and we classified the roles of patent applicants were found in the knowledge-flow network.

**Conclusions:**

Our results showed the organizations that are leading each area of wound-healing technology. Furthermore, from the results, we identified specific institutions that are efficient for spreading knowledge related to wound-healing technology based on the patents. This information can contribute to the planning of investment strategies and technology policies related to wound-healing.

## Introduction

The wound-care market is estimated to be worth $6.7 billion worldwide, and it is projected to grow rapidly over the next ten years. The growth of the market is related to the increasing number of chronic-wound patients [1]. In the United States, approximately 6.5 million people suffer from chronic wounds, and US$25 billion is spent annually on providing appropriate therapy. This financial burden is growing rapidly because of the aging population and the sharp rise in the number of patients with diabetes and obesity, which has contributed to a surge in the number of patients with chronic wounds worldwide [2,3,4]. Diabetes and obesity can result in an increased incidence of ulcerations such as leg or foot ulcers, which require wound treatment over their lifetime, as well as exorbitant medical expenses [5,6]. However, while the need for wound-healing has increased, there has also been a rapid increase in the development of cost-effective wound-healing technologies [7,8].

Diverse wound-healing technologies that are suitable for each type of wound condition have been developed. The primary wound-healing technologies include traditional dressings, antimicrobial dressings, anti-inflammatory and analgesic dressings, wound-drug delivery, advanced dressings containing biological or naturally derived agents, medicated sutures, and tissue-engineered skin substitutes. In addition, hyperbaric oxygen, negative pressure wound therapy (NPWT), and laser-wound-healing are among the primary wound-healing technologies, and are considered as relatively advanced technologies. Each wound-healing technology has been reviewed in detail in medical literature [7,9]. However, there have been few quantitative studies into trends associated with these technologies, which include the latest advanced wound-healing technologies. Furthermore, there has been limited focus on the institutions that play a key role in the development of each wound-healing technology as well as the knowledge flow related to wound-healing technology among those institutions. An understanding of this information can help the decision making of participants in the growing wound-care market with respect to the planning of investment strategies and technology policies [10,11,12]. We therefore analyzed the trends associated with wound-healing technologies and knowledge flow in the wound-healing industry using patent data.

As a fundamental knowledge resource, patent data plays an important role in identifying technology development trends [13,14,15]. Patent analysis is used frequently to analyze the competition in technological changes at an industry or national level, to evaluate the technological strengths and weaknesses of competitors, and to examine the potential of foreign markets. In addition, patent analysis can also contribute to the forecasting of future trends regarding technology or a specific industry [16,17,18]. Therefore, for our trend analysis, we used patent data related to wound-healing technologies.

First, we discuss the trends related to wound-healing patents based on a preliminary analysis. Next, we extract topics related to wound-healing technologies in the abstracts of those patents by applying the structural topic model (STM). The patent applicants are divided into four types of groups, namely firms, research institutes, universities, and individuals, and we performed an STM analysis using group information as covariates. Based on these processes, we examined the different kinds of wound-healing technologies that may or may not have been considered by the four applicant groups up to the present. We also extracted top lists of active patent applicants for the selected topics. We performed patent citation analyses for applicants in order to examine the network structure of the wound-healing industry in terms of knowledge flows about patents [19,20,21]. Finally, to identify the role of each applicant in the knowledge-flow network, we used k-means clustering in terms of the centralities of the knowledge-flow network.

In this study, we focus on the wound-healing patents applications that were made at the United States Patent and Trademark Office (USPTO), China Patent and Trademark Office (CPTO), European Patent Office (EPO), and Japan Patent Office (JPO) from January 1, 1972 to December 31, 2015.

This research is organized as follows. In section 2, we briefly review existing wound-healing technologies. In section 3, we perform a preliminary analysis of wound-healing patents, while in section 4 and 5, we describe the results of the STM and citation analysis. Finally, in section 6, we summarize the results of this research and propose possible developments.

## A brief review of wound-healing technologies

There has been a gradual improvement in the wound-dressing technologies considering the diverse wound conditions in existence, and several studies have reviewed these technologies [7,22]. In particular, Boateng and Catanzano [7] reviewed various wound-healing technologies considering the ingredients used in dressing products, and their roles in the healing of various types of wound conditions. This section consists of four major parts for explaining the Boateng and Catanzano [7]’s review about wound-healing technologies: ingredients to be used for wound-dressing material, wound-dressing technologies, materials to be used for wound-healing, and advanced wound-healing therapies and physical therapies.

Ingredients to be used for wound-dressing materials are broadly classified as natural inert polymers, natural bioactive polymers, and synthetic polymers. Natural inert polymers have outstanding biocompatibility and biodegradability as substances with sources that are plants, bacterial, fungal, or animals. In the case of natural bioactive polymers, it includes components that form part of the body matrix, and it helps the physiological activity as part of the natural wound-healing process. Furthermore, synthetic polymers have good moisture-absorption capacity and can help to maintain a moist wound environment. In particular, sodium alginate, collagen, hyaluronic acid, and chitosan parts of natural bioactive polymers have been used for wound-dressing materials because of their effectiveness at creating a good wound-healing environment.

Several wound-dressing technologies have been used for wound-healing as traditional dressings, wound dressings for drug delivery, antimicrobial dressings, and anti-inflammatory and analgesic dressings. In traditional dressings, cotton, wool, bandages, and gauzes are mostly used. This is still a common approach because of their ease of use, accessibility to most surgical hospitals, and low cost. Wound dressings for drug delivery are also used to transport substances such as antimicrobials, anti-inflammatory agents, and analgesics for wound-healing, and materials such as hydrogel, hydrocolloids, foams, films, and wafers can be used to transport the substances. In the case of antimicrobial dressings, they are used to kill bacteria or fungi that are present in infected wounds, and can reduce the risks of reinfection during wound-healing, surgical procedures, or when changing the dressing. Finally, anti-inflammatory and analgesic dressings can help to reduce the pain and inflammation in wounded areas.

Examples of materials that are used for wound-healing include advanced dressings containing biological agents, dressings containing naturally derived agents, and tissue-engineered skin substitutes. Advanced dressings containing biological agents include growth factor, nucleic acids, and stem cells, all of which accelerate the regeneration of cells at the site of a wound or adjust the level of substances to be produced when natural wound-healing occurs. Dressings that contain naturally derived agents can be obtained in nature, and include naturally occurring plant compounds and honey. These substances are used to heal wounds and burns. Finally, tissue-engineered skin substitutes can be used as substitutes for skin because they can help recolonization of fibroblast and keratinocytes.

Finally, examples of wound-healing therapy technologies include advanced wound-healing therapies and physical therapies. Advanced wound-healing therapies also include oxygen-associated therapies and NPWT. Oxygen-associated therapies are helpful in patients with diabetes-related foot ulcers, and they reduce the risk of amputation. NPWT reduces the likelihood bacterial infection, and increases the blood flow under the skin using subatmospheric pressure. Furthermore, the application of physical therapies to wound-healing accelerates the natural wound-healing process using an electric current or laser.

## Wound-healing patent data

We obtained patent data regarding wound-healing technologies from the WISDOMAIN. The WISDOMAIN offers patent data that were submitted to KIPO, USPTO, CPTO, JPO, and EPO. Therefore, we obtained from the USPTO, CPTO, JPO, and EPO patent data that include English abstracts. Patent data pertaining to wound-healing as at February 2, 2016 was obtained by performing a search of keywords presented in Table 1. Furthermore, from the obtained data, we omitted patent data that were unrelated to wound-healing based on IPC code and removed replicated patent data by the reapplication of the same technology. We obtained 3,253 patents that had been submitted to the USPTO, 4,814 to the CPTO, 2,229 to the EPO, and 1,000 to the JPO.

**Table 1:** Keywords related to wound-healing technology

Keywords search
(wound*) and (dressing* or repair* or healing* or therapeutic or therapy or therapies or "care" or "cares" or "treat" or "treats" or treating* or treatment* or remedy or remedies or "cure" or "cures") and (chronic or burn* or sore* or ulcer* or tissue* or bioactive* or polymer* or alginate* or collagen* or synthetic* or hyaluronic* or chitosan* or hydrogel* or cotton* or wool* or bandage* or gauze* or hydrocolloid* or foam* or film* or wafer* or antibiotic* or silver* or antimicrobial* or inflammatory or analgesic* or nucleic* or stem or honey or aloe or extract* or suture* or oxygen or negative or electrical or electromagnetic or laser*)

**Fig 1:**
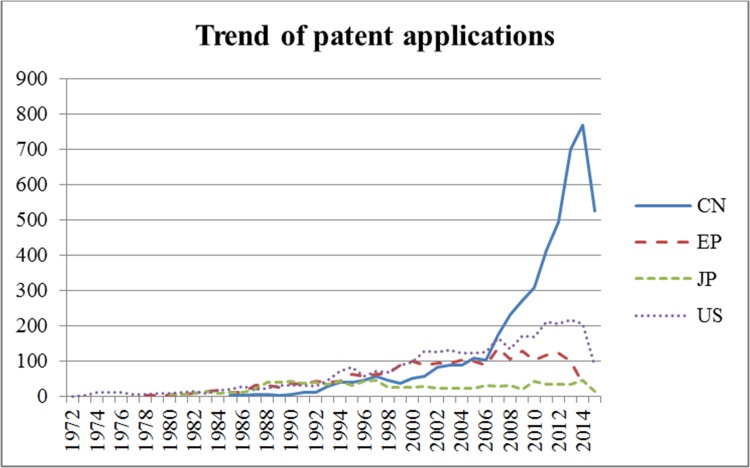
Time series showing number of patent applications received by trademark offices

Fig 1. shows the trends in the number of patent applications submitted at four large patent offices from 1972 to 2015. In the case of JPO, the number of patent applications made has remained constant since 1988. For EPO, the number of patent applications has increased marginally since 1988, and the number of applications at USPTO increased more rapidly than at EPO. On the other hand, in the case of CPTO, the number of applications increased fastest since 2006. In particular, since 2008, the number of applications at CPTO exceeded the number at USPTO. This is believed to be because of the rapid growth in the need for wound-healing technologies in China.

Table 2 shows the nationalities of the applicants at the USPTO, CPTO, EPO, and JPO. In the case of the USTPO, CPTO, and JPO, local applicants are seen to have the highest frequency, while for the EPO, applicants of the US have the highest frequency. Across the globe, applicants from the US, China, and Japan have assumed leading positions in the area of wound-healing patents, followed by applicants from Germany and the United Kingdom. One unusual finding is that Chinese inventors do not usually register their patents in other countries.

**Table 2:** Nationalities of applicants to patent and trademark office

USPTO		CPTO		EPO		JPO	
Nationality	Number	Nationality	Number	Nationality	Number	Nationality	Number
US	1,384	CN	3,289	US	950	JP	664
DE	125	US	371	DE	255	US	185
GB	128	DE	89	GB	214	DE	34
JP	75	JP	39	JP	89	GB	26
SE	53	SE	37	SE	63	CH	17
TW	50	KR	36	FR	47	TW	13
KR	37	CH	20	CH	42	KR	7
CH	34	FR	18	DK	41	IT	6
FR	34	DK	15	IT	36	FR	5
DK	29	AU	14	NL	36	CA	4
Other	285	Other	102	Other	364	Other	39
Missing	1,019	Missing	784	Missing	92	Missing	0
Sum	3,253	Sum	4,814	Sum	2,229	Sum	1,000

Table 3 shows the top five applicants at each patent office. In the USPTO, CPTO, and EPO, both KCI and Smith & Nephew, which are firms in the medical industry, were highly ranked. At the JPO, local firms were usually highly ranked, while in the case of CPTO, unlike other patent offices, the top applicants included several universities in China. One unusual observation is that Shiseido is one of the top applicants for wound-healing patents, although its major business is not related to medicine.

**Table 3:** Top five applicants at each patent office

USPTO		CPTO		EPO		JPO	
Applicants	No. of applied patents	Applicants	No. of applied patents	Applicants	No. of applied patents	Applicants	No. of applied patents
KCI	57	KCI	34	Smith & Nephew	57	Terumo Corp	29
Smith & Nephew	56	Zhejiang University	25	Johnson & Johnson	47	Sekisui Chemical	24
3M	36	Smith & Nephew	21	KCI	45	Shiseido	21
Johnson & Johnson	35	Human Genome Sciences	21	Paul Hartmann A. G.	33	Noevir	18
Tyco Healthcare	23	Donghua University	21	3M	32	Tyco Healthcare	16

We confirmed the proportion of grant / application for the top 15 applicants without any distinction of the patent offices (Table 4). Smith & Nephew, KCI, Johnson & Johnson, Human Genome Sciences, and Paul Hartmann A. G. were ranked as the top 5 applicants based on the number of applications. Furthermore, KCI, Johnson & Johnson, and Bristol-Myers Squibb Company showed a high proportion of grant / application of over 80%. On the other hand, Zhejiang University applied for 24 patents but was not granted any patents for protecting the rights.

**Table 4:** Top 15 applicants who applied for several patents

Applicant	No. of applied patents	No. of granted patents	Grant / application proportion (%)
Smith & Nephew	75	48	64.0
KCI	62	52	83.9
Johnson & Johnson	56	45	80.4
Human Genome Sciences	42	25	59.5
Paul Hartmann A. G.	36	18	50.0
3M Innovative Properties Company	35	26	74.3
Tyco Healthcare	32	16	50.0
Terumo	29	8	27.6
Zhejiang University	24	0	0.0
Sekisui Chemical	24	5	20.8
Ethicon	24	17	70.8
Coloplast AS	24	17	70.8
Beiersdorf AG	24	17	70.8
Molnlycke Health Care AB	21	16	76.2
Bristol-Myers Squibb Company	21	17	81.0

Next, we classified wound-dressing patents according to the first four digits of the IPC codes. Table 5 shows the top 10 IPC codes in each patent office. The wound-dressing patents are mostly associated with the A61K code (Preparations for medical, dental, or toilet purposes) in all patent offices. Next, with the exception of the USPTO, the A61L (Methods or apparatuses for sterilizing materials or objects in general; disinfection, sterilization, or deodorization of air; chemical aspects of bandages, dressings, absorbent pads, or surgical articles; materials for bandages, dressings, absorbent pads, or surgical articles) and A61F (Filters implantable into blood vessels; prostheses; devices providing patency to, or preventing the collapse of tubular structures of the body) rank second and third, respectively. In the case of the USPTO, the ranks of A61F and A61L are reversed compared to other patent offices. We also observed that IPC sections A and C are the main categories of wound-dressing patents. Section A is related to human necessities, while Section C is related to chemistry and metallurgy.

**Table 5:** Distribution of IPC main classes by trademark office

USPTO		CPTO		EPO		JPO	
IPC	Number	IPC	Number	IPC	Number	IPC	Number
A61K	1,231	A61K	2,550	A61K	606	A61K	415
A61F	636	A61L	863	A61L	501	A61L	141
A61L	290	A61F	306	A61F	326	A61F	102
A61M	226	A61M	202	C12N	132	C07K	51
A61B	164	A61B	120	A61M	130	C12N	43
A01N	101	C12N	81	C07K	72	A61M	42
A61N	91	C07K	55	A61B	70	A61B	39
C07K	74	A61N	54	C07D	50	C07D	16
C12N	58	A23L	47	A61N	41	A61N	12
C07D	36	A01N	43	A01N	37	C09J	9

Furthermore, we classified wound-dressing patents according to full IPC codes in order to identify the number of patents based on a detailed classification (Table 6). The IPC code that was counted the most is A61F-013/00, followed by A61L-015/28 and A61F-013/02. These are presented as apparatuses, consumables, or ingredients for wound-healing.

**Table 6:** Distribution of top 10 IPC classes (Source: WIPO)

IPC code	Number	Definition
A61F-013/00	409	Bandages or dressings (suspensory bandages A61F 5/40; radioactive dressings A61M 36/14); Absorbent pads (chemical aspects of, or use of materials for, bandages, dressings or absorbent pads A61L 15/00, A61L 26/00)
A61L-015/28	242	Polysaccharides or their derivatives
A61F-013/02	236	Adhesive plasters or dressings (A61F 13/06-A61F 13/15 take precedence; surgical adhesives or cements A61L 24/00)
A61K-035/78	165	Materials from plants
A61K-036/898	161	Liliaceae (Lily family), e.g. daylily, plantain lily, Hyacinth or narcissus
A61M-001/00	145	Suction or pumping devices for medical purposes; Devices for carrying-off, for treatment of, or for carrying-over, body-liquids; Drainage systems (catheters A61M 25/00; tube connectors, tube couplings, valves or branch units, specially adapted for medical use A61M 39/00; devices for taking samples of bloodA61B 5/15; saliva removers for dentists A61C 17/06; filters implantable into blood vessels A61F 2/01)
A61L-015/44	110	Medicaments
A61K-036/889	110	Arecaceae, Palmae or Palmaceae (Palm family), e.g. date or coconut palm or palmetto
A61M-027/00	103	Drainage appliances for wounds, or the like (implements for holding wounds open A61B 17/02)
A61K-009/70	95	Web, sheet or filament bases

## Text mining using STM

STM provides a useful way of incorporating “metadata” associated with the text into the analysis using document-level covariates. As covariates for the “metadata,” we can apply information such as where the text was written, who wrote it, and characteristics of the author. Therefore, it allows analysts to understand the relationships between metadata and topics in their text corpus [23,24].

In this research, for the covariates, we classified applicants of wound-healing patents into the following four groups: firms, research institutes, universities, and individuals. Free-standing and non-profit institutes were included in the research institutes group; university-associated institutes were included in the university group, and we used STM to explain the topics on which each applicant group has focused with respect to wound-healing technologies.

We summarized the abstracts of 8,178 wound-healing patents using 10,269 terms arising from data preprocessing such as parsing, stemming, and stopword removal. Next, to conduct the STM analysis, we determined a suitable number of topics by increasing the number of topics from five to 15. Based on our results, the most suitable number of topics was 9, because keywords within each topic explained the topic most clearly at 9. Furthermore, we named each topic based on keywords that are related to wound-healing technologies. We referred to literature in order to name wound-healing technologies [7,22]. The results of topic clustering and naming are shown in Table 7. We also determined the number of patents classified into more than one topic based on the probability that each patent belongs to each topic using the result of STM analysis. As a result, 5,354 patents were included in one topic; 1,474 patents were included in two topics; 444 patents were included in three topics; 906 patents were included in more than four topics. In addition, the number of patents related to each topic is shown in Table 8.

**Table 7:** Keywords and naming by each topic

Topic	Keywords and naming	
1	Keywords	Prepare, solution, material, chitosan, water, agent, wound, acid, obtain, dress, medic, gel, fiber, collagen, comprise, antibacterial, can, composite, weight, use
	Naming	Antibacterial material including chitosan or collagen
2	Keywords	Body, portion, can, medic, end, arrange, part, suture, surface, side, open, bag, fix, patient, wound, tube, operation, use, device, inner
	Naming	Device for wound-healing
3	Keywords	Disease, compound, treat, wound, pharmaceutical, treatment, use, ulcer, present, disorder, include, chronic, inflammatory, agent, relative, topical, diabetes, heal, comprise, therapeutic
	Naming	Topical treatment using pharmacological agent
4	Keywords	Cell, tissue, use, wound, acid, growth, factor, protein, active, heal, agent, polypeptide, present, also, culture, peptide, contain, human, promote, include
	Naming	Growth factor
5	Keywords	Part, medicine, Chinese, traditional, effect, prepare, root, blood, material, treat, weight, promote, raw, pain, follow, radix, disclose, remove, relieve, wound
	Naming	Traditional Chinese medicine
6	Keywords	Wound, pressure, device, treatment, system, negative, include, tissue, fluid, control, use, connect, source, apparatus, may, oxygen, mean, therapy, member, one
	Naming	Negative pressure wound therapy (NPWT)
7	Keywords	Wound, layer, dress, material, adhesive, film, comprise, surface, polymer, form, absorb, one, least, use, foam, fabric, include, may, sheet, cover
	Naming	Film for dressing
8	Keywords	Wound, burn, medicine, oil, treat, scald, powder, effect, prepare, use, ointment, treatment, scar, can, cure, medical, surface, pain, extern, time
	Naming	Dressing material for healing burn
9	Keywords	Skin, wound, heal, can, effect, promote, extract, use, prepare, tissue, application, function, component, active, product, repair, drug, reduce, infection, ingredient
	Naming	Dressing material for healing infection

**Table 8:** Number of applied patents classified into more than one topic

No. of applied patents classified into one topic: 5354
→ Topic 1: 758, Topic 2: 330, Topic 3: 516, Topic 4: 783, Topic 5: 765, Topic 6: 614, Topic 7: 997, Topic 8: 348, Topic 9: 243
No. of applied patents classified into two topics: 1474
→ Topic 5&8: 425, Topic 1&7: 190, Topic 3&4: 141, Topic 2&6: 115, Topic 6&7: 89, Topic 2&7: 80, Topic 4&9: 79, Topic 1&9: 60, Topic 8&9: 51, Topic 3&9: 39, Topic 4&7: 35, Topic 1&4: 31, The others: 139
No. of applied patents classified into three topics: 444
No. of applied patents classified into more than four topics: 906

In the STM, we calculate the “expected topic proportion,” which refers to the extent of the relation between a topic and a text corpus. Therefore, we calculated the “expected topic proportion” for each group, and we then extracted from each group the top five topics for which the “expected topic proportion” is high (Table 9).

**Table 9:** Top five topics by each applicant group

Applicant group	No. of patents	No. of applicants	First	Second	Third	Forth	Fifth
Firm	3,749	2,185	Topic 7	Topic 4	Topic 6	Topic 1	Topic 3
Research institute	238	164	Topic 1	Topic 4	Topic 7	Topic 9	Topic 3
University	804	373	Topic 1	Topic 4	Topic 9	Topic 3	Topic 7
Individual	2,079	1,903	Topic 5	Topic 8	Topic 9	Topic 2	Topic 1

From the results obtained, while the focus topics of the research institutes and universities were similar, the results for the firms and individuals showed a different tendency. The firms applied primarily for wound-healing patents that are related to Topic 7 (*Film for dressing*). In the case of the research institutes and universities, the applications pertained primarily to Topic 1 (*Antibacterial material including chitosan or collagen*), and in the case of the individuals, applications were related primarily to Topic 5 (*Traditional Chinese medicine*). Furthermore, in addition to these topics, Topic 4 (*Growth factor*) was a popular topic common to firms, research institutes, and universities.

**Fig 2:**
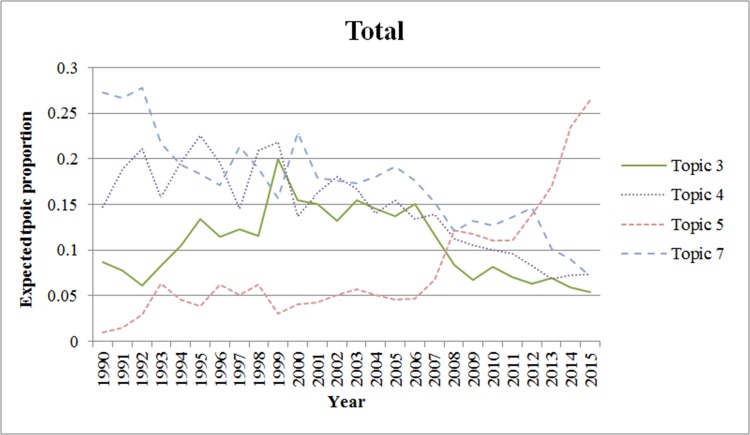
Trends of wound-healing technologies.

**Fig 3:**
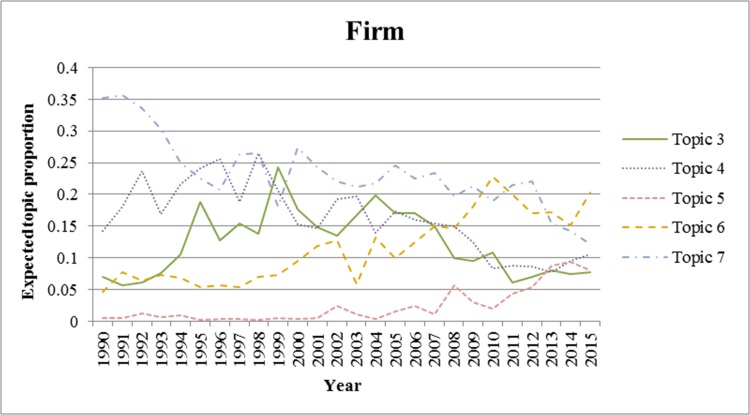
Trends of wound-healing technologies for firms.

**Fig 4:**
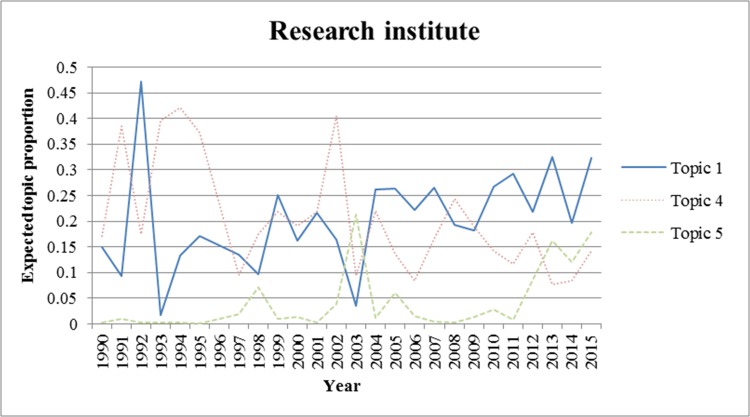
Trends of wound-healing technologies for research institutes.

**Fig 5:**
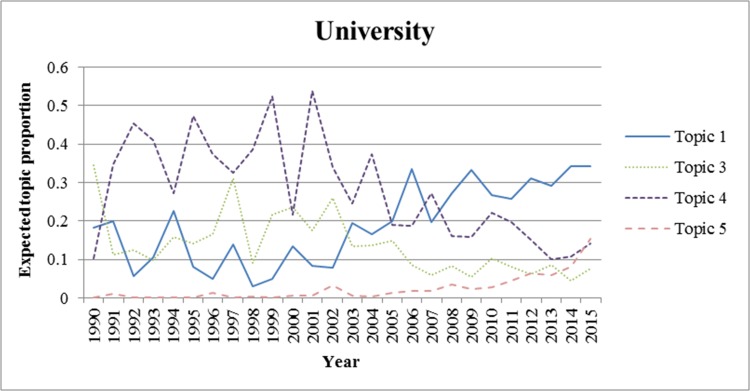
Trends of wound-healing technologies for universities.

**Fig 6:**
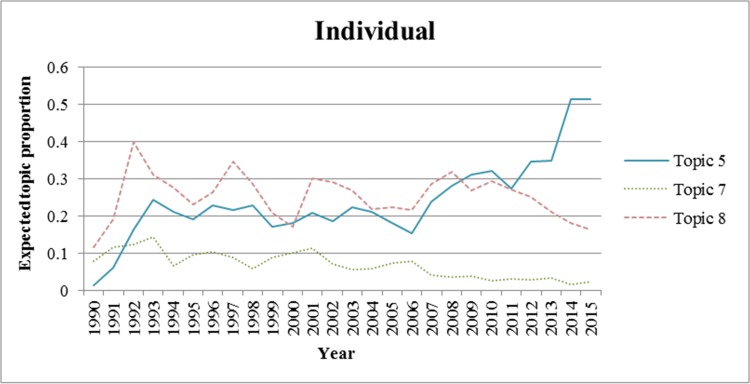
Trends of wound-healing technologies for individuals.

We also analyzed the trends for each topic from 1990 to 2015. We excluded the period prior to 1990 because there were few applications for wound-healing patents during this period. In Figs 2–6., we show the trends of wound-healing technologies in terms of the total number of applicants and each applicant group, and we plotted only topics that have upward or downward trends in the figures. In these figures, the x-axis refers to the year, and the y-axis refers to the expected topic proportion, which is the relative frequency of occurrence among topics in each year.

According to the results of the trend analysis, Topic 5 *(Traditional Chinese medicine)* was a popular topic for which the frequency of occurrence increased for all applicant groups. Other topics had different tendencies in terms of the frequency of occurrence by each applicant group. In terms of the overall applicants, the frequencies of occurrence of Topic 4 (*Growth factor*) and Topic 7 (*Film for dressing*) decreased gradually. The frequency of occurrence of Topic 3 (*Topical treatment using pharmacological agent*) increased steadily until 1999, and decreased from 2000. In the case of firms, the trend for each topic, except for Topic 6, was very similar to that of the overall patent applications. The frequency of occurrence of Topic 6 (*NPWT*) generally increased until 2010, decreased from 2011, and rebounded again in 2014. Furthermore, in the case of research institutes, there was a high proportion of patent applications for Topic 4, and it decreased from 2002 to 2012; however, it has been increasing again since 2013. The frequency of occurrence of Topic 1 (*Antibacterial material including chitosan or collagen*) has generally increased since 2003. In the case of universities, the frequency of occurrence of Topic 1 has been increasing steadily, while for Topics 3 and 4, it has been decreasing since 2001 and 2002, respectively. Finally, in the case of individuals, the frequency of occurrence of Topic 8 was high in general, and that of Topic 7 has been decreasing since 2001.

We examined the kinds of applicants that focused on different kinds of topics by matching each patent with the topic having the highest expected frequency of occurrence, and we extracted some top applicants for each topic, as shown in Table 10. Based on the results, the number of applications for wound-healing patents varied for each topic. The patent applications that are related to Topic 6 (*NPWT*) and Topic 7 (*Film for dressing*) accounted for a large proportion of all patent applications made by the top applicants. On the other hand, patent applications that were related to Topic 2 (*Device for wound-healing*) and Topic 8 (*Dressing material for healing burn*) accounted for a relatively small proportion of all patent applications by the top applicants.

**Table 10:** Few top applicants for each topic and the number of patents applied for by applicants

**Topic 1** (*Antibacterial material including chitosan or collagen*)
1. Donghua University: 18 2. Zhejiang University: 15 3. Johnson & Johnson: 14 4. Sichuan University: 12
**Topic 2** (*Device for wound-healing*)
1. Covidien LP: 4 2. Suzhou AND Science & Technology Development: 3 3. Second Military Medical University: 3 4. Jiangsu Province Jian'erkang Pharmaceutical Surgical Dressing: 3 5. Suzhou Ruihua Hospital: 3 6. The Fourth Military Medical University of the Chinese People's Liberation Army: 3
**Topic 3** (*Topical treatment using pharmacological agent*)
1. Amgen: 16 2. Warner-Lambert Company: 16 3. The Upjohn Company: 15 4. American Cyanamid: 11 5. Pfizer: 10
**Topic 4** (*Growth factor*)
1. Human Genome Sciences: 38 2. Sekisui Chemical: 19 3. The Regents of the University of California: 10 4. Actamax Surgical Materials: 8
**Topic 5** (*Traditional Chinese medicine*)
1. Qingdao Municipal Hospital: 7 2. Shandong Provincial Hospital: 4 3. Cui Hefang: 4 4. Baijia Kangjian (Tianjin) Biotechnology: 4
**Topic 6** (*Negative pressure wound therapy*)
1. KCI: 47 2. Smith & Nephew: 28 3. Tyco Healthcare: 23 4. Paul Hartmann A. G.: 17 5. Covidien LP: 12
**Topic 7** (*Film for dressing*)
1. Smith & Nephew: 43 2. Johnson & Johnson: 32 3. 3M Innovative Properties Company: 31 4. Terumo Corp: 19 5. Coloplast AS: 18
**Topic 8** (*Dressing material for healing burn*)
1. Northwest A&F University: 3 2. Hou Qinying: 3
**Topic 9** (*Dressing material for healing infection*)
1. Noevir: 13 2. Shiseido: 9 3. Lion: 7 4. Pola Chem: 4

**Fig 7:**
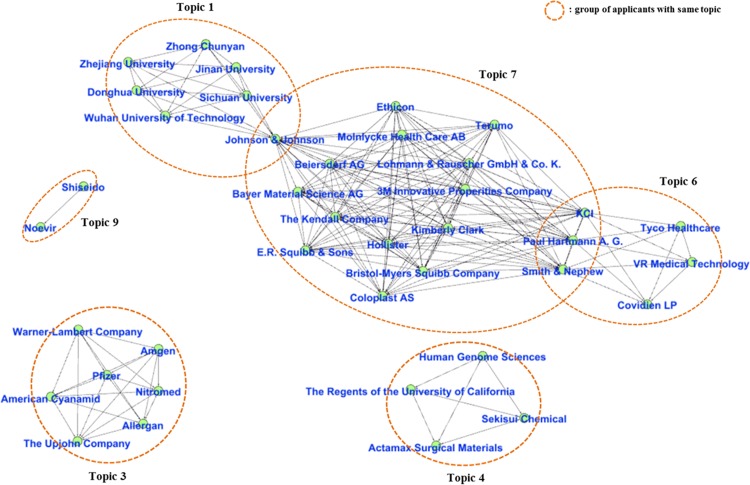
Co-topic network.

We also extracted applicants that applied for more than 8 wound-healing patents for each topic, and we demonstrated the result as a network by connecting the extracted applicants related to the same topic (Fig 7). In the network, the nodes refer to each applicant, and the edges refer to two applicants that focused on the same topic. The network shows the topics on which each applicant was focused. We note that some applicants focused on two topics. Johnson & Johnson focused on topics 1 and 7, while KCI, Paul Harfmann A.G., and Smith & Nephew focused on Topics 6 and 7.

## Citation analysis and knowledge flow

**Fig 8:**
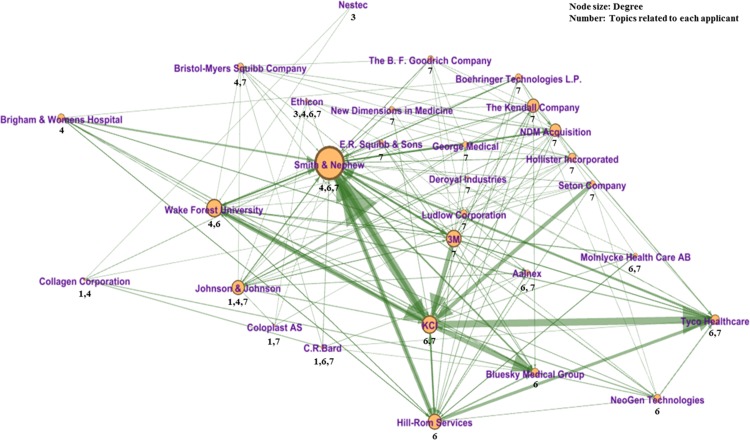
Knowledge networks in the wound-healing ecosystem.

We conducted a social network analysis (SNA) to analyze the knowledge flow among some applicants of wound-healing patents. We obtained 3,776 sets of backward citation information for all wound-healing patents and extracted a total of 29 applicants that have a high degree of relatively in the network. Next, we presented the knowledge network about the patents for the 29 applicants using their backward citation information. In the network, each node refers to the applicant, and the direction of the arc indicates the direction of knowledge flow. If a patent of applicant A cites a patent of applicant B, the direction of knowledge flow goes from B to A. Furthermore, the width of the arc indicates the number of citations. Fig 8. shows the knowledge flow between 29 nodes. We marked the topics for which each applicant has a high concentration under each node. Therefore, this citation network indicates the knowledge flows, with respect to patents, between applicants associated with each topic area.

We confirmed that there is usually a knowledge flow about patents in each topic area. As a result, the extent of the knowledge flow varied with the topic area. The knowledge flow was relatively active within the areas of Topic 6 (*NPWT*) and Topic 7 (*Film for dressing*). On the other hand, the knowledge flow was relatively weak for the areas of the other topics.

**Fig 9:**
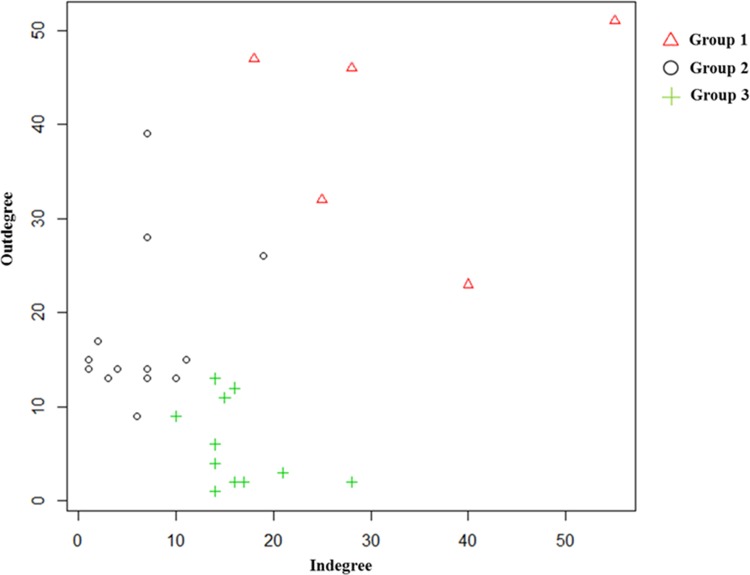
Distribution of each applicant in knowledge network.

The degree of centrality is suitable for examining the local centrality as a measure of the number of direct links to other nodes. The degree of centrality is also divided into indegree centrality and outdegree centrality. The indegree centrality is determined by counting the number of inflow connections, whereas the outdegree centrality is determined by counting the number of outflow connections [25]. Therefore, we can use these to determine the roles of each node in the knowledge network [20]. We conducted a k-means clustering analysis in order to classify each applicant into three groups based on the values of the indegree centrality and outdegree centrality following Lee [20]’s method. Fig 9. illustrates the distribution of each applicant in the knowledge network.

As summarized in Table 11, the three classified applicant groups play different roles in the knowledge network of the wound-healing industry. Group 1 has a high outdegree and indegree, and plays a keystone role in network. In Group 1, there were 3M, Johnson & Johnson, KCI, and Smith & Nephew as the main firms that applied for several patents relatively. Group 2 has a relatively higher outdegree compared to the indegree, and it plays the role of distributing the knowledge. Coloplast AS and Molnlycke Health Care AB were the main firms in Group 2. Finally, Group 3 has a relatively higher indegree compared to the outdegree, and it plays the role of receiving pure knowledge. Bristol-Myers Squibb Company, Ethicon, and Tyco Healthcare were the main firms in Group 3.

**Table 11:** Classifications of each applicant according to their roles

Group	Classification	Number of applicants	Applicants	Description
1	Knowledge keystone players	5	3M, Johnson & Johnson, KCI, Smith & Nephew, Wake Forest University	High outdegree and indegree
2	Knowledge-distributing mediators	13	C.R.Bard, Collagen Corporation, *Coloplast AS*, E.R. Squibb & Sons, Hill-Rom Services, Hollister Incorporated, *Molnlycke Health Care AB*, NDM Acquisition, NeoGen Technologies, New Dimensions in Medicine, Seton Company, The B. F. Goodrich Company, The Kendall Company	Relatively higher outdegree compared to the indegree
3	Pure knowledge receivers	11	Aalnex, Bluesky Medical Group, Boehringer Technologies L.P., Brigham & Womens Hospital, *Bristol-Myers Squibb Company*, Deroyal Industries, *Ethicon*, George Medical, Ludlow Corporation, Nestec, *Tyco Healthcare*	Relatively higher indegree compared to the outdegree

## Discussion

The worldwide increase in the number of patients diagnosed with chronic wounds is driving the extensive development of wound-healing technologies tailored for particular wound conditions. In this paper, we used STM and co-topic network analyses to examine this technological trend by focusing on different groups of related patent applicants: firms, universities, research institutes, and individuals.

The STM analysis identified 9 topics related to wound-healing technologies. For firms, the most popular topic was *Film for dressing*. The patent applications from universities and research institutes were related primarily to *Antibacterial material including chitosan or collagen*. *Traditional Chinese medicine* was the major topic of interest to individuals. We also analyzed the pattern of each group’s emphasis on wound-healing technology. *Traditional Chinese medicine* was a popular topic whose frequency of occurrence increased for all groups. The patterns of the other topics were less consistent and differed from group to group.

It is important to establish the flow between various institutions of knowledge related to wound-healing technology when planning investment strategies and technology polices. Therefore, we used the citation network to examine this knowledge flow, and we identified the role of each institution using k-means clustering. We confirmed that the extent of the knowledge flow for each topic area varied considerably. *Negative-pressure wound therapy* and *film for dressing* were the active topics in relation to patent citations, whereas *antibacterial material including chitosan or collagen*, *topical treatment using pharmacological agent*, and *growth factor* generated very few citations. Our k-means clustering identified each group according to its role in the network and showed that only a few firms are knowledge keystones. As such, applicants such as KCI, Smith & Nephew, Johnson & Johnson, and 3M dominate the wound-healing market.

Our results showed the organizations that are leading each area of wound-healing technology. Furthermore, the results determined specific institutions that are efficient for spreading knowledge related to wound-healing technology with respect to patents. This information can contribute to the planning of investment strategies and technology policies that are related to wound-healing. For example, KCI is highly efficient for spreading the knowledge and for investing because it is a knowledge keystone in the citation network.

In our study, we examined the knowledge flows between the applicants and the trends of wound-healing technologies with respect to patents. However, we could not confirm that such patents were used for the commercialization of products. Therefore, which patents related to wound-healing technology are used for what kinds of products need to be searched and analyzed. Furthermore, firms can adopt a strategy that divides patents into these categories for preventing copying technologies and commercialization of products. For instance, KCI adopted the strategy of mono-product; therefore, they must have maintained many patents to prevent copying technologies related to mono-product. Similarly, what patent strategies are adopted by firms in the wound-healing industry need to be analyzed. These will be a part of future work.
